# Association Between Posttraumatic Stress Disorder and Mortality Among Responders and Civilians Following the September 11, 2001, Disaster

**DOI:** 10.1001/jamanetworkopen.2019.20476

**Published:** 2020-02-05

**Authors:** Ingrid Giesinger, Jiehui Li, Erin Takemoto, James E. Cone, Mark R. Farfel, Robert M. Brackbill

**Affiliations:** 1 World Trade Center Health Registry, New York City Department of Health and Mental Hygiene, Long Island City, New York

## Abstract

**Importance:**

Posttraumatic stress disorder (PTSD) has been associated with increased mortality, primarily in studies of veterans. The World Trade Center Health Registry (Registry) provides a unique opportunity to study the association between PTSD and mortality among a population exposed to the World Trade Center attacks in New York, New York, on September 11, 2001 (9/11).

**Objectives:**

To assess whether 9/11-related probable PTSD (PTSD) is associated with increased mortality risk, as well as whether this association differs when including repeated measures of PTSD over time vs a single baseline assessment.

**Design, Setting, and Participants:**

A longitudinal cohort study of 63 666 Registry enrollees (29 270 responders and 34 396 civilians) was conducted from September 5, 2003, to December 31, 2016, with PTSD assessments at baseline (wave 1: 2003-2004) and 3 follow-up time points (wave 2: 2006-2007, wave 3: 2011-2012, wave 4: 2015-2016). Data analyses were conducted from December 4, 2018, to May 20, 2019.

**Exposures:**

Posttraumatic stress disorder was defined using the 17-item PTSD Checklist–Specific (PCL-S) self-report measure (score ≥50) at each wave (waves 1-4). Baseline PTSD was defined using wave 1 PCL-S, and time-varying PTSD was defined using the PCL-S assessments from all 4 waves.

**Main Outcomes and Measures:**

Mortality outcomes were ascertained through National Death Index linkage from 2003 to 2016 and defined as all-cause, cardiovascular, and external-cause mortality.

**Results:**

Of 63 666 enrollees (38 883 men [61.1%]; mean [SD] age at 9/11, 40.4 [10.4] years), 6689 (10.8%) had PTSD at baseline (responders: 2702 [9.5%]; civilians: 3987 [12.0%]). Participants who were middle aged (2022 [12.5%]), female (3299 [13.8%]), non-Latino black (1295 [17.0%]), or Latino (1835 [22.2%]) were more likely to have PTSD. During follow-up, 2349 enrollees died (including 230 external-cause deaths and 487 cardiovascular deaths). Among all enrollees in time-varying analyses, PTSD was associated with all-cause, cardiovascular, and external-cause mortality, with adjusted hazard ratios (AHRs) of greater magnitude compared with analyses examining baseline PTSD. Among responders, time-varying PTSD was significantly associated with increased risk of all-cause (AHR, 1.91; 95% CI, 1.58-2.32), cardiovascular (AHR, 1.95; 95% CI, 1.25-3.04), and external-cause (AHR, 2.40; 95% CI, 1.47-3.91) mortality. Among civilians, time-varying PTSD was significantly associated with increased risk of all-cause (AHR, 1.54; 95% CI, 1.28-1.85), cardiovascular (AHR, 1.72; 95% CI, 1.15-2.58), and external-cause (AHR, 2.11; 95% CI, 1.06-4.19) mortality.

**Conclusions and Relevance:**

The risk of mortality differed in examination of baseline PTSD vs repeated measures of PTSD over time, suggesting that longitudinal data should be used where possible. Comparable findings between responders and civilians suggest that 9/11-related PTSD is associated with an increased mortality risk.

## Key Points

**Question:**

What is the association of mortality with baseline and repeated assessments of posttraumatic stress disorder in a population exposed to the World Trade Center attacks on September 11, 2001, over 13 years of follow-up?

**Findings:**

In this cohort study of 63 666 World Trade Center Health Registry enrollees, posttraumatic stress disorder was associated with an increased risk of mortality and associations were strengthened when considering posttraumatic stress disorder status over time compared with a single posttraumatic stress disorder assessment at study entry.

**Meaning:**

Without considering the time-varying outcomes of posttraumatic stress disorder, important differences in the association of posttraumatic stress disorder and mortality may be masked.

## Teaser

This cohort study assesses whether posttraumatic stress disorder following the World Trade Center attacks in New York, New York, on September 11, 2001, is associated with all-cause, cardiovascular, and external-cause mortality among responders and civilians.

## Introduction

Posttraumatic stress disorder (PTSD), a life-changing mental health condition, occurs due to exposure to traumatic events, such as natural or human-made disasters, war, or violence. Posttraumatic stress disorder symptoms may persist long after the incident traumatic event,^1,2^ and wide-ranging, long-term effects of PTSD on morbidity have been described.^3,4,5,6,7,8,9^ Evidence for the association between PTSD and mortality comes primarily from veterans studies, with PTSD assessed at 1 time point. Excess mortality in the 30 years following the Vietnam War was first described among Vietnam service veterans.^10^ Subsequent studies in this population and others supported the initial findings: PTSD increased the risk of all-cause, cardiovascular, and external-cause mortality.^11,12,13,14^

Among those exposed to the World Trade Center (WTC) attacks in New York, New York, on September 11, 2001 (9/11), the estimated prevalence of probable PTSD ranges from 3.8% to 29.6%, depending on the population.^15^ In a recent mortality study among enrollees of the WTC Health Registry (Registry), standardized mortality ratios for suicide were significantly elevated among responders vs the reference population.^16^ In previous mortality studies among Registry enrollees, inconclusive findings on the association between exposure to 9/11 and mortality were observed,^16,17^ potentially owing to insufficient follow-up time.

The Registry tracks long-term physical and mental health consequences among a large, diverse cohort of responders and civilians exposed to 9/11 and its aftermath.^18^ Although overall mortality among Registry enrollees is lower compared with the general population, potentially increased cause-specific mortality^16,17^ is a concern among this population, particularly in relation to PTSD.^19,20,21^ The present study has 2 aims: to assess whether 9/11-related PTSD measured repeatedly over the life course of enrollees following exposure is associated with an increased risk of mortality (all-cause, cardiovascular, and external-cause) over the follow-up period; and in order to compare with existing literature, to assess the association of 9/11-related PTSD measured 2 to 3 years after exposure and subsequent risk of mortality.

## Methods

### Data Source

More than 71 000 individuals who met the Registry inclusion criteria participated in the 2003-2004 baseline interview (wave 1 [W1]) with sections on demographic characteristics, WTC disaster exposure, and health information as described elsewhere.^18,22^ Approximately 17% coverage of the estimated eligible exposed population, including rescue and recovery workers and volunteers, Lower Manhattan residents, area workers, passersby, and students and school staff from the catchment area were enrolled.^18^ Of all enrollees, 30% were identified and enrolled through lists provided by employers, government agencies, and other entities (list-identified), and 70% responded to an outreach campaign (self-identified). To date, three follow-up surveys have been administered, wave 2 ([W2], 2006-2007), wave 3 ([W3], 2011-2012), and wave 4 ([W4], 2015-2016). The survey response rates among those aged 18 years or older at the time of survey were 67.6% at W2, 65.8% at W3, and 53.8% at W4.^23,24^ The reporting of this study followed the Strengthening the Reporting of Observational Studies in Epidemiology (STROBE) reporting guideline for cohort studies. The institutional review boards at the Centers for Disease Control and Prevention and the New York City Department of Health and Mental Hygiene (NYC DOHMH) approved the Registry protocol; NYC DOHMH approved this study. Verbal informed consent was obtained from all participants. Participants do not receive financial compensation.

### Mortality 

Vital status was ascertained through National Death Index linkage, from January 1, 2003, through December 31, 2016. National Death Index search results were processed by the Registry through algorithm and manual review of potential matches, as described elsewhere.^25^ The underlying cause of death (*International Statistical Classification of Diseases and Related Health Problems, 10th Revision*, death codes) was obtained from the linked National Death Index records and classified as all-cause, cardiovascular, or external-cause mortality (eTable in the Supplement).

### Posttraumatic Stress Disorder 

Probable PTSD (hereafter, PTSD) was defined using the PTSD Checklist–Specific (PCL-S) at each wave (W1-W4). The PCL-S consists of 17 Likert items, corresponding to the 3 PTSD symptom clusters from the *Diagnostic and Statistical Manual of Mental Disorders, Fourth Edition* (*DSM-IV*),^26^ and assesses the extent to which 9/11 event-specific symptoms bothered respondents in the past 4 weeks. The PCL-S is a well-validated measure with good temporal stability, internal consistency (α > 0.75), test-retest reliability (correlation coefficient, *r* = 0.66), and high convergent validity (*r* = 0.58-0.93).^27^ In the present study, the internal consistency was excellent (Cronbach α = 0.94). A total symptom severity score was calculated by summing responses to the 17 items, with responses scored from 1 (not at all) to 5 (extremely). Posttraumatic stress disorder was defined as a PCL-S score of 50 or above to increase specificity and minimize false-positives.^28^

For aim 1, we allowed PTSD to vary based on the PCL-S assessments at each wave (hereafter, time-varying PTSD). For aim 2, PTSD was based only on the W1 PCL-S assessment (hereafter, baseline PTSD). These definitions of PTSD are not based on health care professional diagnoses or aligned with formal diagnostic criteria. Instead, the definitions are survey-based classifications that capture the varying burden of PTSD symptoms over time among enrollees.^27^

### Covariates

Covariates were selected a priori following literature review and included self-reported demographic characteristics and relevant comorbidities. Demographic characteristics reported at W1 included age, sex, marital or cohabitating status, race/ethnicity, and smoking history. A 4-level socioeconomic status variable was derived using W1 household income and educational attainment: high (income ≥$50 000, any level of education), intermediate (income <$50 000, college graduate or higher), low (income <$50 000, high school graduate or less), and missing. A count of W1 self-reported conditions (asthma, angina, hypertension, myocardial infarction, heart disease, other heart condition, stroke, emphysema, and diabetes) was derived (0, 1, 2, ≥3) to provide an estimate of baseline disease burden. Mental health condition before 9/11 was defined (yes or no) if enrollees reported having pre-9/11 depression, anxiety, or other emotional problems, excluding PTSD. Responders were considered workers and volunteers involved in rescue, recovery, cleanup, or other disaster-related activities at the WTC site and/or at the Staten Island recovery operations or on transport barges for at least 1 shift between 9/11 and June 30, 2002.^29^ Civilians included residents, students enrolled, or school staff who worked south of Canal Street, as well as area workers and passersby present south of Chambers Street in Manhattan on 9/11. Source of enrollment classified enrollees as self-identified (telephone or website opt-in) or list identified (eg, employer lists).

### Analytic Sample

We excluded enrollees who died before follow-up (n = 161), withdrew (n = 1101), had insufficient information for a National Death Index match (n = 356), had a missing birth date (n = 19), were younger than 18 years on 9/11 (n = 3073), were older than 65 years at enrollment (n = 2683), were W1 proxy respondents (n = 207), or had W1 PCL-S item nonresponse with no further survey completion (n = 160), resulting in an analytic sample of 63 666 enrollees. The sample of 63 666 included 1981 enrollees with incomplete PCL-S responses at W1 but who completed the PCL-S in at least 1 follow-up survey.

### Statistical Analysis

Pearson χ^2^ tests were used to assess differences in the study sample by baseline PTSD. To assess whether those with PTSD were at an increased risk for mortality (all-cause, cardiovascular, and external-cause mortality), adjusted hazard ratios (AHRs) and 95% CIs were calculated through Cox proportional hazards regression, using time on study as the time scale. All deaths, regardless of cause, were considered in the all-cause mortality models. For cause-specific models, only deaths from the underlying cause of interest were considered; if an enrollee died of another cause, they contributed person-time from enrollment until their date of death, at which point they were censored, assuming independent censoring.^30^ In addition, given known differences in morbidity and mortality risk, all multivariable analyses were calculated overall and stratified by enrollee group.^18,31,32^

To assess the time course of PTSD, we performed aim 1 analysis in a full sample of 63 666, using extended Cox proportional hazards models.^30^ An extended Cox model conducts a separate Cox analysis for each time window using the specific value of the time-dependent variable (PTSD) at the beginning of that time window.^33^ Then, the extended Cox proportional hazards model calculates a proportionately weighted mean of all time window-specific results, resulting in a single AHR estimate for PTSD.^30,33,34^ Person-years were calculated from the date of enrollment until the earliest of the date of death, loss to follow-up, or end of follow-up (December 31, 2016). Loss to follow-up was defined by missing a study wave and not returning to complete any subsequent wave (last date of wave response period was used as exit date).

In aim 2 analysis, a conventional Cox proportional hazards model was used to examine the association of mortality with baseline PTSD; person-years were calculated from the date of enrollment to the earliest of date of death or end of follow-up (December 31, 2016). Analysis 2 was conducted among 61 685 enrollees who had baseline PTSD data available, excluding 1981 individuals who provided incomplete PCL-S responses at W1.

To further understand the association between PTSD and suicide, a subanalysis of both external mortality aims was repeated, limiting to deaths due to suicide. Asian race was collapsed to a multiracial, other, or unknown group owing to small cell sizes.

We assessed the functional form of age with cumulative martingale residual plots. The proportional hazards assumption was tested using time interaction terms for all covariates with the natural log of time, generated in an extended Cox model. When the proportional hazards assumption was violated, the product term with time was included. Statistical analyses were performed from December 4, 2018, to May 20, 2019, using SAS, version 9.4 (SAS Institute Inc). Significance was set at a 2-sided *P* < .05.

### Sensitivity Analysis

Deaths occurring within the first 2 years of follow-up may be unrelated to PTSD, which may have a delay between onset and effect and mortality.^14,35,36^ The latency period of PTSD’s influence on mortality is unknown and may have wide variation. To explore possible delayed or increased association of PTSD on deaths occurring later in the follow-up period, we lagged our follow-up start time by 2 years (excluded deaths and all person-years in first 2 years) and repeated aim 1 analyses (sensitivity 1).^37^ To address missing data for income at W1 (10.5% missing) and missing or incomplete PCL-S at all 4 waves (3%-7% missing), aim 1 analysis was repeated using multiply imputed data (sensitivity 2). Wave 1 income and W1 to W4 PCL-S scores were imputed through fully conditional specification methods using SAS MI procedure (n = 10 with 100 burn iterations; income fully conditional specification logistic, PCL-S: fully conditional specification regression).^38^ Imputation models included primary analysis variables (including vital status) and auxiliary variables. The PCL-S score was imputed chronologically for each wave; if an enrollee did not complete a particular wave, the PCL-S score was not imputed.

## Results

A total of 63 666 enrollees were included in this study; of these, 38 883 (61.1%) were men, 40 848 participants (64.2%) were non-Latino white, 36 072 participants (56.7%) reported never smoking, 39 177 (61.5%) reported a household income of $50 000 or more, and the mean (SD) age at 9/11 was 40.4 (10.4) years. Overall, 6689 participants (10.8%) reported PTSD at baseline (responders: 2702 [9.5%], civilians: 3987 [12.0%]); middle-aged (2022 [12.5%]), female (3299 [13.8%]), non-Latino black (1295 [17.0%]), and Latino (1835 [22.2%]) participants were more likely to have PTSD (Table 1).

**Table 1.** Distribution of Selected Study Characteristics and PTSD at Baseline of Study Sample^a^

Characteristic	No. (%)			
	Total^b^	PTSD^c^	No PTSD^c^	*P* Value^d^
Total	63 666	6689	54 996	
Age at 9/11, y				
<25	4306 (6.8)	341 (8.1)	3895 (91.9)	<.001
25-34	15 334 (24.1)	1332 (8.9)	13 672 (91.1)	
35-44	21 052 (33.1)	2365 (11.6)	18 097 (88.4)	
45-54	16 774 (26.3)	2022 (12.5)	14 109 (87.5)	
≥55	6200 (9.7)	629 (10.7)	5223 (89.3)	
Mean (SD)	40.4 (10.4)	41.5 (9.8)	40.1 (10.4)	
Sex				
Female	24 783 (38.9)	3299 (13.8)	20 585 (86.2)	<.001
Male	38 883 (61.1)	3390 (9.0)	34 411 (91.0)	
Married or cohabitating^e^				
Yes	40 406 (63.5)	3578 (9.1)	35 878 (90.9)	<.001
No	22 434 (35.2)	3047 (14.0)	18 730 (86.0)	
Race/ethnicity				
Non-Latino				
White	40 848 (64.2)	2863 (7.2)	37 159 (92.8)	<.001
Black	7879 (12.4)	1295 (17.0)	6323 (83.0)	
Latino	8516 (13.4)	1835 (22.2)	6444 (77.8)	
Asian	3943 (6.2)	368 (9.9)	3345 (90.1)	
Multiracial, other, and unknown	2480 (3.9)	328 (16.0)	1725 (84.0)	
SES (income and education)				
Income ≥$50 000, all educational levels	39 177 (61.5)	2850 (7.4)	35 629 (92.6)	<.001
Income <$50 000, college or higher education	11 422 (17.9)	1846 (16.6)	9277 (83.4)	
Income <$50 000, high school graduate or less	6315 (9.9)	1490 (24.8)	4507 (75.2)	
Missing as category	6752 (10.6)	503 (8.3)	5583 (91.7)	
Enrollee group				
Civilians	34 396 (54.0)	3987 (12.0)	29 205 (88.0)	<.001
Responders	29 270 (46.0)	2702 (9.5)	25 791 (90.5)	
Smoking status^e^				
Never	36 072 (56.7)	3516 (10.0)	31 655 (90.0)	<.001
Former or current	27 044 (42.5)	3131 (11.9)	23 195 (88.1)	
Pre-9/11 history of depression, anxiety, or emotional problem				
No	58 461 (91.8)	5764 (10.2)	50 903 (89.8)	<.001
Yes	5205 (8.2)	925 (18.4)	4093 (81.6)	
Self-reported health conditions, No.^f^				
0	42 375 (66.6)	4025 (9.8)	37 082 (90.2)	<.001
1	16 219 (25.5)	1837 (11.7)	13 871 (88.3)	
2	3745 (5.9)	560 (15.5)	3049 (84.5)	
≥3	1327 (2.1)	267 (21.2)	994 (78.8)	
Source of enrollment				
List-identified	18 998 (29.8)	1524 (8.4)	16 727 (91.6)	<.001
Self-identified	44 668 (70.2)	5165 (11.9)	38 269 (88.1)	

Abbreviation: PCL-S, PTSD Checklist–Specific; PTSD, posttraumatic stress disorder; SES, socioeconomic status.

^a^ Probable PTSD considered as PCL-S score of 50 or higher.

^b^ Includes participants with incomplete PCL-S scores at baseline (n = 1981).

^c^ Row percentages were calculated with those missing PTSD excluded from the denominators.

^d^ *P* values were calculated for PTSD vs no PTSD.

^e^ Missing data at baseline: marital status (n = 826) and smoking status (n = 550).

^f^ Includes asthma, emphysema, angina, hypertension, myocardial infarction, heart disease, other heart conditions, stroke, and diabetes.

During 13 years of mortality follow-up, a total of 2349 deaths occurred among 63 666 enrollees. Of the 230 external-cause deaths, suicide (81 [35.2%]) and accidental poisonings (58 [25.2%]) were most common. Among 487 with cardiovascular-associated deaths, the majority of individuals (362 [74.3%]) died of ischemic heart diseases.

### Aim 1: Time-Varying PTSD and Mortality

Time-varying PTSD and mortality outcomes among all enrollees are reported in Table 2. Responders with PTSD had a nearly 2-fold increased risk of all-cause (AHR, 1.91; 95% CI, 1.58-2.32) and cardiovascular (AHR, 1.95; 95% CI, 1.25-3.04) mortality. Responders with PTSD had a 2.4-fold increase in the risk of external-cause mortality (AHR, 2.40; 95% CI, 1.47-3.91). In civilians with PTSD, we observed a 54% higher risk of all-cause mortality (AHR, 1.54; 95% CI, 1.28-1.85), 72% higher risk of cardiovascular mortality (AHR, 1.72; 95% CI, 1.15-2.58), and 2-fold increase in the risk of external-cause mortality (AHR, 2.11; 95% CI, 2.06-4.19).

**Table 2.** Mortality and Time-Varying PTSD Stratified by Enrollee Group, World Trade Center Health Registry, 2003-2016

Enrollee Group	Mortality					
	All-Cause		Cardiovascular		External-Cause	
	HR (95% CI)	*P* Value	HR (95% CI)	*P* Value	HR (95% CI)	*P* Value
Deaths, No./total No.^a^	1516/63 666		288/63 666		137/63 666	
**All Enrollees (N = 63 666; Person-Years at Risk, 653 720)**						
Time-varying PTSD						
Unadjusted	1.89 (1.67-2.14)	<.001	2.10 (1.59-2.77)	<.001	2.60 (1.78-3.81)	<.001
Adjusted^b^	1.71 (1.50-1.96)	<.001	1.83 (1.35-2.46)	<.001	2.29 (1.54-3.41)	<.001
**Responders (n = 29 270; Person-Years at Risk, 365 318)**						
Time-varying PTSD						
Unadjusted	2.06 (1.72-2.47)	<.001	1.95 (1.28-2.97)	<.001	2.75 (1.72-4.39)	<.001
Adjusted^c^	1.91 (1.58-2.32)	<.001	1.95 (1.25-3.04)	.003	2.40 (1.47-3.91)	<.001
**Civilians (n = 34 396; Person-Years at Risk, 430 638)**						
Time-varying PTSD						
Unadjusted	1.76 (1.48-2.09)	<.001	2.24 (1.55-3.23)	<.001	2.44 (1.26-4.69)	.01
Adjusted^c^	1.54 (1.28-1.85)	<.001	1.72 (1.15-2.58)	.01	2.11 (1.06-4.19)	.03

Abbreviations: HR, hazard ratio; PTSD, posttraumatic stress disorder.

^a^ Total deaths reflect the entire sample not stratified by enrollee group.

^b^ Adjusted for age, sex, marital status, race/ethnicity, socioeconomic status, smoking, pre-9/11 mental health history, preenrollment health conditions, rescue/recovery worker status, and source of enrollment interview.

^c^ Adjusted for age; sex; marital status; race/ethnicity; socioeconomic status; smoking; pre-9/11 history of depression, anxiety, or emotional problems; health conditions at enrollment; and source of enrollment.

### Aim 2: Baseline PTSD and Mortality

Responders with baseline PTSD had a 63% higher risk of all-cause mortality (AHR, 1.63; 95% CI, 1.36-1.95) and a 2-fold increase in the risk of cardiovascular mortality (AHR, 2.02; 95% CI, 1.39-2.93), while those with baseline PTSD were not at increased risk of external-cause mortality (AHR, 1.24; 95% CI, 0.75-2.05) (Table 3). Civilians with baseline PTSD had a 38% higher risk for all-cause mortality (AHR, 1.38; 95% CI, 1.18-1.60) and a 2.7-fold increase in the risk of external-cause mortality (AHR, 2.73; 95% CI, 1.57-4.75), while associations with cardiovascular mortality were not significant but were in the same direction (AHR, 1.34; 95% CI, 0.98-1.85).

**Table 3.** Mortality and Baseline PTSD Stratified by Enrollee Group, World Trade Center Health Registry, 2003-2016

Enrollee Group	Mortality					
	All-Cause		Cardiovascular		External-Cause	
	HR (95% CI)	*P* Value	HR (95% CI)	*P* Value	HR (95% CI)	*P* Value
Deaths, No./total No.^a^	2237/61 685		470/61 685		211/61 685	
**All Enrollees (n = 61 685; Person-Years at Risk, 771 618)** ^**b**^						
Baseline PTSD						
Unadjusted	1.80 (1.62-2.10)	<.001	2.00 (1.59-2.51)	<.001	1.95 (1.38-2.75)	<.001
Adjusted^c^	1.48 (1.32-1.66)	<.001	1.58 (1.23-2.01)	<.001	1.71 (1.18-2.46)	.004
**Responders (n = 28 493; Person-Years at Risk, 355 783)**						
Baseline PTSD						
Unadjusted	1.85 (1.56-2.19)	<.001	2.18 (1.54-3.09)	<.001	1.52 (0.94-2.47)	.09
Adjusted^d^	1.63 (1.36-1.95)	<.001	2.02 (1.39-2.93)	<.001	1.24 (0.75-2.05)	.41
**Civilians (n = 33 192; Person-Years at Risk, 415 835)**						
Baseline PTSD						
Unadjusted	1.77 (1.53-2.04)	<.001	1.87 (1.38-2.54)	<.001	3.11 (1.87-5.18)	<.001
Adjusted^d^	1.38 (1.18-1.60)	<.001	1.34 (0.98-1.85)	.07	2.73 (1.57-4.75)	<.001

Abbreviations: HR, hazard ratio; PTSD, posttraumatic stress disorder.

^a^ Total deaths reflect the entire sample not stratified by enrollee group.

^b^ Excludes individuals with incomplete PTSD Checklist–Specific scores at baseline (n = 1981).

^c^ Adjusted for age; sex; race/ethnicity; socioeconomic status; smoking; pre-9/11 history of depression, anxiety, or emotional problem; health conditions at enrollment; enrollee group; and source of enrollment.

^d^ Adjusted for age; sex; race/ethnicity; socioeconomic status; smoking; pre-9/11 history of depression, anxiety, or emotional problems; health conditions at enrollment; and source of enrollment.

Baseline PTSD was not associated with suicide among responders (n = 56; AHR, 0.68; 95% CI, 0.24-1.93). However, in time-varying analyses, responders with PTSD were at an increased risk of suicide compared with those without PTSD (n = 34; AHR, 2.47; 95% CI, 1.15-5.31). Owing to too few events and unstable estimates, results for civilians are not presented.

### Sensitivity Analyses

When we lagged study start time by 2 years, compared with the primary analysis, the AHRs were modestly attenuated among responders (AHR for all-cause mortality, 1.65; 95% CI, 1.32-2.05; AHR for cardiovascular mortality, 1.66; 95% CI, 1.01-2.73; AHR for external-cause mortality, 2.01; 95% CI, 1.12-3.58) and slightly increased for civilians (AHR for all-cause mortality, 1.59; 95% CI, 1.31-1.93; AHR for cardiovascular mortality, 1.88; 95% CI, 1.24-2.83; AHR for external-cause mortality, 2.37; 95% CI, 1.11-5.07) (Figure). When we imputed income and PCL-S, AHRs for both responders (AHR for all-cause mortality, 1.95; 95% CI, 1.62-2.34; AHR for cardiovascular mortality, 2.16; 95% CI, 1.42-3.28; AHR for external-cause mortality, 2.34; 95% CI, 1.46-3.75) and civilians (AHR for all-cause mortality, 1.53; 95% CI, 1.28-1.82; AHR for cardiovascular mortality, 1.65; 95% CI, 1.12-2.42; AHR for external-cause mortality, 2.11; 95% CI, 1.05-4.24) were comparable with the primary analyses (Figure).

**Figure.**
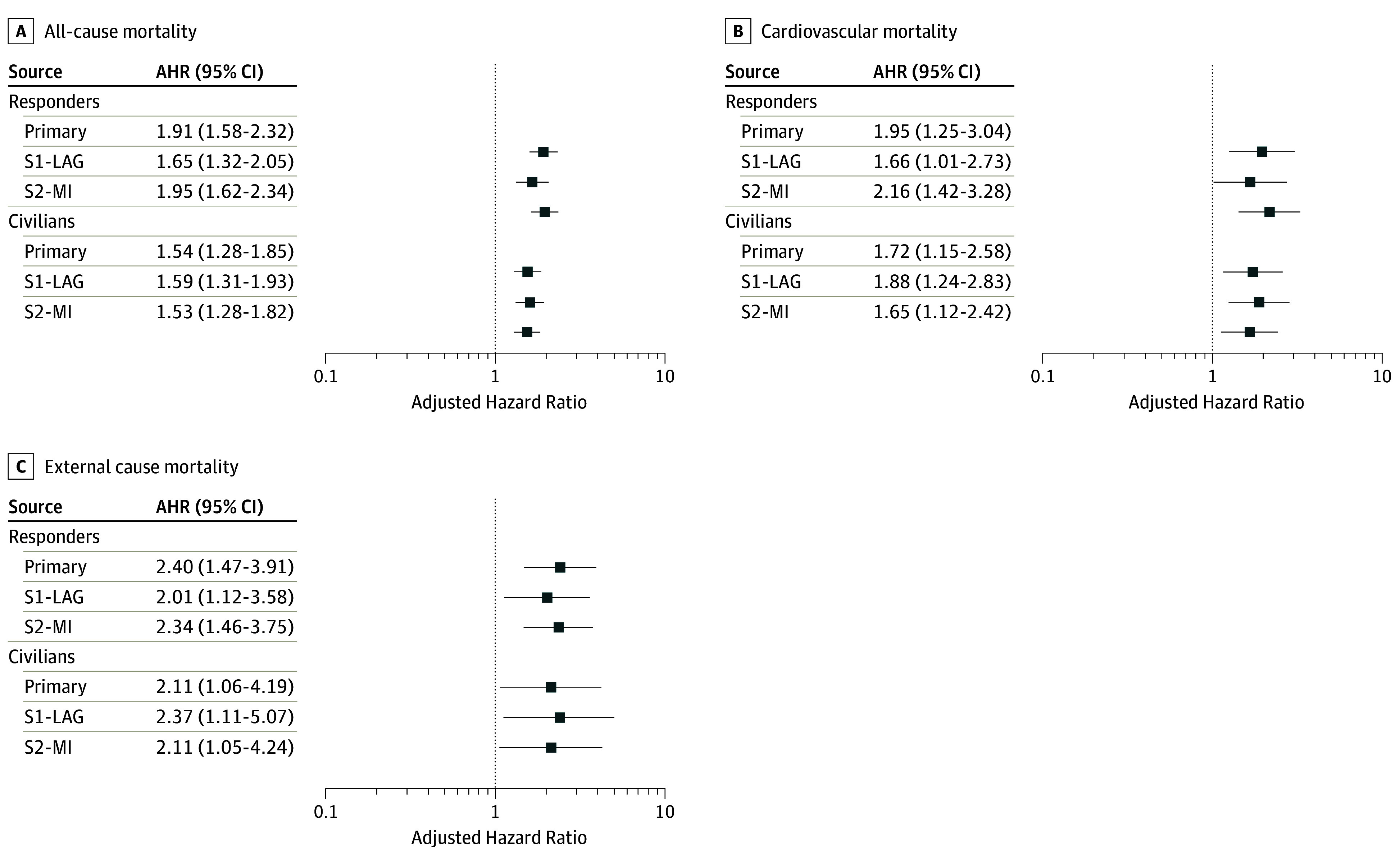
Sensitivity Analysis Results: Cox Proportional Hazards Regression for Time-Varying Posttraumatic Stress Disorder (PTSD) and Mortality Outcomes Stratified by Enrollee Group Outcomes are shown for all-cause mortality (A), cardiovascular mortality (B), and external-cause mortality (C). Data were adjusted for age; sex; marital status; race/ethnicity; socioeconomic status; smoking; pre-9/11 history of depression, anxiety, or emotional problems; health conditions at enrollment; and source of enrollment. AHR indicates adjusted hazard ratio; S1-LAG, sensitivity 1: lagged mortality (2005-2016); S2-MI, sensitivity 2: multiple imputed PTSD Checklist scores at waves 1 to 4, and income at wave 1. Error bars indicate 95% CI.

## Discussion

We found that 9/11-related PTSD appears to be associated with an increased risk of all-cause, cardiovascular, and external-cause mortality in this diverse 9/11-exposed population, based on analyses of both a single measurement of baseline PTSD in 2003-2004 and repeated measures of PTSD over 13 years of follow-up. Mortality risk differed when baseline vs time-varying PTSD was examined. Time-varying PTSD appeared to be associated with increased hazard ratios for mortality across all 3 mortality outcomes in the overall sample, while the magnitude of the association varied when stratified by enrollee group. Our findings suggest the importance of considering changes in PTSD symptoms over time when studying mortality risk factors.

To our knowledge, this study is one of the first to contrast repeated measures of PTSD with a baseline assessment; therefore, the time-varying results are difficult to compare with existing literature. Data that suggest an association between PTSD and mortality come primarily from US studies on veterans,^11,12,13,39,40,41,42,43,44^ with limited studies in other populations.^45,46,47^ Our baseline results are broadly consistent with this literature, which observed an association between baseline PTSD and all-cause,^11,45,46,48^ cardiovascular,^11,13,39^ and external-cause^12,46,47^ mortality. These findings build on previous Registry studies in which PTSD was identified as a risk factor for stroke and a modest association with cerebrovascular disease hospitalization was observed.^19,21^

Our results were inconsistent with those of 2 studies in veteran populations of all-cause mortality^40,42^ and 1 study of cardiovascular mortality,^43^ which found that PTSD and mortality were not statistically significantly associated, with a point estimate near 1.0 after adjusting for comorbidities. Notable differences between these studies may explain the disparate findings, including handling of comorbidities, study design, and study population characteristics. Comorbid depression and PTSD are highly prevalent in the Registry enrollees^49^; postenrollment depression and comorbid health conditions may be on the PTSD-mortality causal pathway and therefore were not controlled for in this study.

Among responders, a heterogeneous population of individuals from unaffiliated sources (eg, volunteers) and affiliated organizations (eg, firefighters, law enforcement),^18^ baseline PTSD was significantly associated with increased risk of all-cause and cardiovascular mortality, but not external-cause mortality. Conversely, time-varying PTSD was significantly associated with all 3 causes of death and the AHR was considerably higher for external-cause mortality. For cardiovascular mortality, these findings build on a recent study among male firefighters, which observed an increased long-term risk of cardiovascular disease.^50^ Although the investigators did not observe PTSD to be a risk factor, they controlled for intermediate conditions between PTSD and cardiovascular disease, and our group of responders was more diverse, including women. It is possible that the unaffiliated responders included in our sample may contribute a different risk profile, and the availability of postdisaster programs to responders, such as routine health screenings, were varied by organization.^51,52^ Additional studies are needed to explore the potential differences among responders, including evaluating and quantifying the contribution of the various health promotion efforts available to specific organizations.

Examination of time-varying PTSD among civilians showed that all 3 mortality outcomes were statistically significant. The cardiovascular mortality risk was 72% higher among civilians with PTSD. For baseline PTSD among civilians, the cardiovascular mortality AHR was not statistically significant. This finding may suggest that the risk for cardiovascular mortality increases with persistent PTSD. Despite differences in demographic characteristics,^18^ prevalence of comorbidities,^53^ and access to monitoring programs^54^ between responders and civilians, a similar mortality risk attributed to PTSD was observed. Although previous literature has demonstrated an association between PTSD and morbidity among civilians,^5,55,56^ to our knowledge, the present study is among the first to demonstrate increased mortality risk among civilians with PTSD.^46^

Multiple mechanisms for the path from PTSD to cardiovascular mortality have been proposed. Potentially modifiable behaviors that are associated with PTSD^4,57^ are considered independent risk factors for mortality^58^ and cardiovascular outcomes.^57,59^ Behavioral factors may mediate the PTSD-cardiovascular pathway. Because this association is not fully attenuated in adjusted models,^60^ there may be additional mechanisms.^6,56^ In addition to behavioral perspective, biological mechanisms have been described,^4,6^ including elevated basal heart rate, which is a strong independent predictor for cardiovascular-related mortality.^61^ Increased cardiovascular demand is described as one plausible physiologic mechanism for the PTSD-cardiovascular disease association,^4^ where long-term increased demand results in systemic inflammation^4,62,63^ and contributes to atherosclerosis through endothelial dysfunction.^4^ Other plausible mechanisms include cellular dysfunction and neuroendocrine activation, which may arise in response to the psychological symptoms of PTSD.^62,64^ Health behaviors^4,6,62^ may confound or mediate the PTSD–cardiovascular disease association, although previous studies suggest that PTSD is an independent risk factor for cardiovascular mortality through a biological demand-capacity framework.^4,5,6,61,62,64^

Despite the increase in suicide rates between 1999 and 2016 in the United States,^65^ death due to suicide is rare, making it difficult to study. Nevertheless, an increased risk of suicide has been reported.^47^ Posttraumatic stress disorder is associated with suicidal thoughts, tendencies, and attempts,^66,67,68^ as well as external-cause mortality,^11,12^ which captures suicide in addition to broader unnatural causes of death, such as accidents. Previous Registry literature found that comorbid depression with PTSD was highly prevalent^49,69^; comorbid depression significantly increases the odds of suicide^47^ and risk of suicidality^68^ for those with PTSD. Our findings are consistent with the literature and demonstrate the importance of PTSD duration on external-cause mortality risk, particularly among responders. Future in-depth studies of the PTSD-suicide association are needed.

This study has important public health implications. It suggests a PTSD-mortality association outside of veterans. In addition, accounting for repeated measurements of PTSD showed an apparently increased risk of all-cause, cardiovascular, and external-cause mortality comparable in responders and civilians. Improvements in the identification of those at risk of developing PTSD following a traumatic experience and long-term follow-up of those with PTSD may help to mitigate this risk. Main strengths of this study are the large sample, longitudinal data, and inclusion of exposed civilians.

### Limitations

The study has limitations. The 2 aims of our study required different exposure definitions, resulting in contrasting limitations. First, differential loss to follow-up is always a concern in longitudinal studies. Implications of intermittent W1 to W3 survey response have been described in detail.^23^ Briefly, enrollees with intermittent response patterns were more likely to experience PTSD than those who had completed all study waves.^23^ This attrition may introduce bias into our findings. However, using a time-varying approach allows participants to miss a study wave but contribute in later waves, potentially minimizing this bias. Because individuals with PTSD are less likely to remain in the study over time^23,70^ and our calculation of person-years in aim 1 excludes deaths that occurred after loss to follow-up, it is more likely that our results are biased toward the null, underestimating the PTSD and mortality association. In addition, we examined PCL-S item nonresponse using multiple imputed data. Sensitivity analysis results were similar to those of the primary analysis, suggesting that bias related to PCL-S item nonresponse over time was minimal.

Second, owing to the nature of our study design, it is possible that some enrollees who experienced PTSD immediately following the WTC disaster recovered before our first PTSD assessment 2 years after the attacks. In baseline PTSD analyses, enrollees with recovered PTSD would be considered as unexposed. This misclassification would likely bias our results toward the null.

Third, PTSD in this study was assessed through self-reported symptoms reflected in the PCL-S and not a clinical diagnosis. This approach is widely used and the PCL-S has been shown to correlate highly with clinician-administered measures.^27^ This study used a PCL-S score of 50 or higher to increase the specificity in defining the PTSD population, which may minimize potential bias.

Symptoms of PTSD were queried specifically to the 9/11 event, which may limit the generalizability of our findings to populations exposed to different traumas. However, our goal was not to differentiate between the various causes of PTSD, but rather between the downstream consequences of PTSD, regardless of the underlying trauma history.

Fourth, in the cause-specific analysis, deaths due to other causes were treated as censored at the time of the death. This categorization assumes that the competing risks of mortality were independent, which is not testable. Common risk factors were included in all survival models in this study, which may remove the mutual effect on the competing risks. This study focused on the association between PTSD and mortality and was not intended to estimate prevalence; therefore, the cause-specific hazard model assuming independent censoring is appropriate.^71^

## Conclusions

The findings appear to support the existing literature, demonstrating a significant association between 9/11-related PTSD and mortality in a diverse population. Mortality risk appears to be similar in both responders and civilians, regardless of differences that exist between these groups, supporting the dynamic association between PTSD and mortality risk. Our findings suggest that it is critical to sustain long-term efforts to identify and treat those at risk of developing PTSD following a traumatic experience.

## Notes

Supplement. **eTable.** List of *ICD-10* Codes for Cause-Specific Mortality
