# Supplementary material for: Association Between Posttraumatic Stress Disorder and Mortality Among Responders and Civilians Following the September 11, 2001, Disaster
Source: JAMA Netw Open. 2020 Feb 5;3(2):e1920476. doi: 10.1001/jamanetworkopen.2019.20476 (PMC12549136; doi:10.1001/jamanetworkopen.2019.20476)
Supplement: Supplement. — eTable. List of ICD-10 Codes for Cause-Specific Mortality [file jamanetwopen-e1920476-s001.pdf]

Supplementary Online Content

Giesinger I, Li J, Takemoto E, Cone JE, Farfel MR, Brackbill RM. Association between posttraumatic stress disorder and mortality among responders and civilians following the September 11, 2001, disaster. *JAMA Netw Open*. 2020;3(2):e1920476. doi:10.1001/jamanetworkopen.2019.20476

eTable. List of *ICD-10* Codes for Cause-Specific Mortality

This supplementary material has been provided by the authors to give readers additional information about their work.

© 2020 Giesinger I et al. *JAMA Network Open.*

eTable: List of *ICD-10* codes for cause-specific mortality	
Cause specific mortality	ICD-10 Code(s)
Cardiovascular Mortality	
Hypertensive diseases	I10, I11
Ischemic heart diseases	I20-I25
Other Forms of Heart Disease - Heart Failure	I50
Cerebrovascular diseases	I63 & I64
Atherosclerosis	I70
External Causes of Mortality	
Transport Accidents	V01-V99
Accidental Poisoning	X40-X49
Other external Causes of Accidental Injury	W00-W19, X00-X39, X50-X59
Intentional Self Harm	X60-X84
Assault	X85-Y09
Event of Undetermined Intent	Y10-Y34
Legal intervention and operations of war	Y35-Y36

© 2020 Giesinger I et al. *JAMA Network Open.*
